# Information and communication technologies-assisted after-hours work: A systematic literature review and meta-analysis of the relationships with work–family/life management variables

**DOI:** 10.3389/fpsyg.2023.1101191

**Published:** 2023-02-01

**Authors:** Alda Santos, Magda Sofia Roberto, Cláudia Camilo, Maria José Chambel

**Affiliations:** Centro de Investigação em Ciência Psicológica, Faculdade de Psicologia, Universidade de Lisboa, Lisbon, Portugal

**Keywords:** meta-analysis, information and communication technologies, after-hours work, work–family/life conflict, work–family/life balance, work–family/life enrichment, well-being

## Abstract

The phenomenon of information and communication technology (ICT)-assisted after-hours work has led to rising academic interest in examining its impact on workers’ lives. ICT-assisted after-hours work may intrude on the home domain and contribute to higher work–family/life conflict, lower work–family/life balance, or higher work–family/life enrichment (the last one owing to the acquisition of competencies transferable to the home domain). Additionally, owing to cultural and societal differences in gender roles, the relationships between ICT-assisted after-hours work and work–family/life management variables may differ between female and male workers. To analyze the current empirical findings, this study performed a literature review with 38 articles and a meta-analysis with 37 articles. Our findings showed that ICT-assisted after-hours work was positively related to work–family/life enrichment (*r* = 0.335, *p* < 0.001; 95% CI [0.290, 0.406]), but also to work–family/life conflict (*r* = 0.335, *p* < 0.001; 95% CI [0.290, 0.406]). However, neither gender nor pre−/post-COVID significantly affect the relationship between ICT-assisted after-hours work and work–family/life conflict. Finally, future research and implications are discussed.

## 1. Introduction

Information and communication technologies (ICT) are defined as a “diverse set of technological tools and resources used to transmit, store, create, share, or exchange information” (UNESCO, 2022). These include a range of devices that allow access to information and communication, irrespective of time and place, such as computers and mobile communication devices. The benefits for organizations, such as improved access to information, enhanced action speed, and availability, among others, have contributed to their increasing implementation and dependence on technology. However, this pervasiveness, which makes it possible for organizations to contact almost any worker at any time, has become an increasing concern in a fast-changing technological environment when matters from the work domain start intruding in workers’ life domain, namely work-related ICT use after the working schedule. The boundaries between domains, that is, the limits (cognitive, physical, and/or behavioral) that will reflect in individuals’ roles (Ashforth et al., 2000; Kreiner, 2006) may, therefore, be subject to alterations as the work domain interferes in the family/life domain.^1^ For example, a company may repeatedly contact an employee during their day-off with their children for some urgent work, which will require the worker to alternate between their parent–professional roles. Depending on the frequency of such situations, work–personal life boundaries become less defined and more blurred (Desrochers et al., 2005). The issue of blurred boundaries created by ICT use after work hours has been raised for some time (Lewis and Cooper, 1999). Consequently, there has been a growing interest in the impact and consequences of after-hours ICT use on work–family/life management constructs, such as work–family/life conflict, enrichment, and balance.

Work-related ICT use after-hours and work–family/life management, as an area of concern resulting from technology’s steady and rapid advancement and invasion by work-related matters of domains that are becoming increasingly less private, have emerged as areas of growing empirical research. Its relevance is particularly related to the recent events associated with the COVID-19 pandemic that rapidly made teleworking (and working from home during the lockdown period) a worldwide work modality choice for many occupations. Studies are increasingly examining the relationships between work-related ICT use after-hours and work–family/life management: its effects on work–family/life conflict, enrichment, and balance. This rising body of empirical knowledge calls for a systematic review that provides a qualitative analysis of all the studies developed in this area, and the trends that can be identified between work-related ICT use after-hours and work–family/life management variables. We aim to present a systematic review of this research, which, to the best of our knowledge, has not yet been published. Our study, therefore, contributes to both theoretical and practical implications for organizational practices.

Furthermore, it is significant to study the quantitative results of this body of research; consequently, we aim to present a meta-analysis to assess the direct relationships between work-related ICT use after-hours and work–family/life management variables. Recent meta-analytic studies have focused on work-related ICT use and well-being (Baumeister et al., 2021) and negative outcomes (ill-being, workload, and so on; Karimikia et al., 2020). However, we consider that there remains a need to explore the relationships between work-related technology after-hours use and work–family/life variables using meta-analytic methodology. This meta-analysis will further contribute to our knowledge of the strength and direction of the effects reported in the empirical research.

Additionally, when exploring events related to both work and home/family domains, it is significant to consider the differences in gender roles attributed to cultural values and beliefs (Wood and Eagly, 2010), which continue to manifest in our society (UN Women, 2022). This recent report that gathers data collected among 20 UN countries shows that although gender equality is almost consensual in theory, attitudes toward gender roles remain linked to the traditional roles concerning work and family. The conventional female role as primary family caregiver may result in a higher conflict between work-related after-hours ICT use, compared to the traditional bread-winner male role. Moreover, empirical literature shows contradictory findings regarding gender differences in the work–family/life field, which calls for further investigation (Shockley et al., 2017). To the best of our knowledge, research on the moderating role of gender in the relationship between after-hours work-related ICT use and work–family/life management variables is limited. Our study attempts to contribute to this area.

## 2. Theoretical background

With organizations resorting to the use of technology, management of roles associated with work and family/life has, therefore, acquired another factor that may contribute to its complexity. Traditionally, managing work and family/life roles has been analyzed from a negative perspective regarding the number of roles that may generate conflict for the individual, and how they may translate into work–family conflict (Greenhaus and Beutell, 1985). Work–family/life conflict was conceptualized as “a form of inter-role conflict in which the role pressures from the work and family domains are mutually incompatible in some respect” (Greenhaus and Beutell, 1985, p: 77). Work–family/life conflict was based on a scarcity viewpoint that assumed that finite resources, such as time and energy, invested in one domain would be lacking in the other domain. In this definition, the direction from the work domain to the family/life domain was considered an interference by the work role in the family/life role. However, literature has come to consider bidirectionality—the family domain may also interfere in the work domain—because of technological advancements and the possibility of being connected to one’s family (and personal life) from the office.

However, a more optimistic framework was developed beyond the scarcity viewpoint considering that domains could form an alliance from which the experiences in one domain could be useful for the other (Greenhaus and Powell, 2006). This role accumulation (Voydanoff, 2001) contributes to work–family/life enrichment, which is defined as “the extent to which experiences in one role improve the quality of life in the other role” (Greenhaus and Powell, 2006, p: 72). In fact, the literature has shown that work and family/life roles offer diverse experiences that can combine and contribute to greater well-being of the individual (developmental aspect), or distress in one domain could be compensated by the role played by the other domain (affective aspect; Greenhaus and Powell, 2006). Additionally, there can be a constructive transference of positive experiences and results from one domain to the other, which potentiates resources (efficiency aspect) and is considered by Greenhaus and Powell (2006) as the most significant characteristic of work–family/life enrichment. As with work–family/life conflict, work–family/life enrichment is bidirectional, with mutually influencing domains.

However, do the absence of work–family/life conflict and presence of work–family/life enrichment inform us whether an individual experiences a balance between these domains? Carlson et al. (2006) explored the definition of work–family/life balance, mostly considered as the lack of conflict and the existence of facilitation between the two domains. The conceptualization was extended as “accomplishment of role related expectations that are negotiated and shared between an individual and his/her role related partners in the work and family/life domains” (Grzywacz and Carlson, 2007, p: 458). Furthermore, work–family/life balance was shown to be a distinct construct from work–family/life conflict and work–family/life enrichment and revealed incremental variance beyond that shown by these constructs for organizational and outcome variables (Carlson et al., 2009). In our study, we opted to include work–family/life conflict, enrichment, and balance, due to their theoretical background, established definitions, and measures (Beigi et al., 2019) as work–family/life management variables.

A significant aspect of work–family/life management is gender roles, which reflect cultural and societal values and beliefs related to the female and male roles played in the work and home domains (Wood and Eagly, 2010). Household chores are still not equally shared, with women assuming the most load compared to men (Cerrato and Cifre, 2018), although some authors claim that gender roles are evolving and becoming more similar, with for example women’s increased academic status and men’s growing interest in balancing work and family lives (Powell et al., 2019). Besides, in a recent meta-analytic study by Shockley et al. (2017), no significant differences were found between genders related to work–family/life conflict. However, the authors call for further research on this subject. In effect, a recent report including 20 countries has shown that, if any, the COVID-19 pandemics consequences on attitudes toward gender roles have been generally negative and led to a regression to traditional stereotypes, such as, in times of crisis and shortage, women should devote themselves to unpaid care duties and abdicate employment in favor of men (UN Women, 2022). Another report showed that the gendered effects of COVID-19, albeit considered to be short-lived, also highlighted inequalities between men and women in the labor market and the division of care among parents (Mesiäislehto, 2022). Therefore, with our focus on the home domain, the potential interference of after-hours work-related technology use is significant to examine possible gender differences in this respect, due to differentiations in female and male roles previously mentioned.

With the occurrence of the COVID-19 pandemics in 2020 and onward, one of the emergency measures to protect workers and allow organizations to continue to work during this crisis was the resource to teleworking. In this case, due to mandatory lockdown during diverse time periods in 2020 in most countries, work was literally transferred to the home domain, at least concerning the occupations that were able to be performed remotely, and most workers had to quickly adapt to this new working mode. Evidently, this was possible due to technology use, and it also created the conditions to a greater potential intrusion of work in the home domain, particularly if it involved longer working hours or work contacts outside of working hours. Therefore, our study also explores the possible differences due to this variable, comparing results from studies developed before and during COVID-19.

## 3. The present study

Our study’s aims include:

- Systematic literature review to summarize empirical research exploring the use of technology (e.g., smartphones, personal computers, tablets, etc.) for work purposes, outside of regular working schedules, and the relationship with work–family/life management variables (conflict, balance, and enrichment)
- Meta-analysis to provide: evidence about effect sizes of after-hours work-related ICT use and work–family/life management; to explore the role of gender in the relationships between after-hours work-related ICT use and work–family/life management; and to test the possible moderating impact of the COVID-19 pandemics on the previously mentioned relationships.

## 4. Materials and methods

### 4.1. Eligibility criteria

Eligibility criteria included studies with the following characteristics: empirical studies (i.e., theoretical articles were excluded), quantitative studies (i.e., qualitative studies were not considered), published in peer-refereed journals (i.e., book chapters, dissertations, theses, conference presentations, and gray literature were not included), samples composed of working participants (i.e., research among non-working participants were not included), work–family/life management variables (namely work–family/life conflict, enrichment, and/or balance) and work-related after-hours ICT use behaviors (studies’ scales were analyzed to ensure that they assessed self-reported work-related after-hours technology use behaviors), and accessibility to the articles was possible.

### 4.2. Data collection

**Figure 1:**
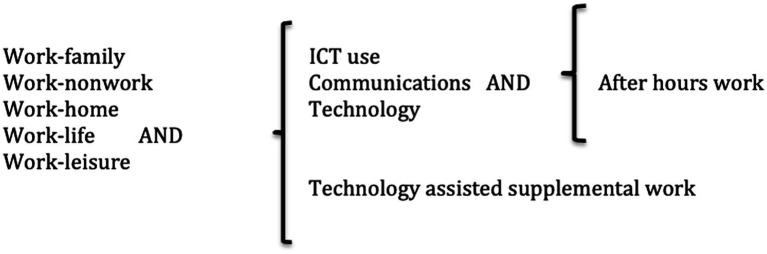
Search words and combinations.

We conducted our search in the digital databases Scopus and Web of Science (all selected bases) using several combinations of search words, as graphically presented in Figure 1. We aimed to select every published article that included work–family/life variables, technology use behaviors, and after-hours of work. We opted not to set time limits in searches and publications date from May 2006 to May 2022. Additionally, no language filters were included, although the search returned only articles written in English.

**Figure 2:**
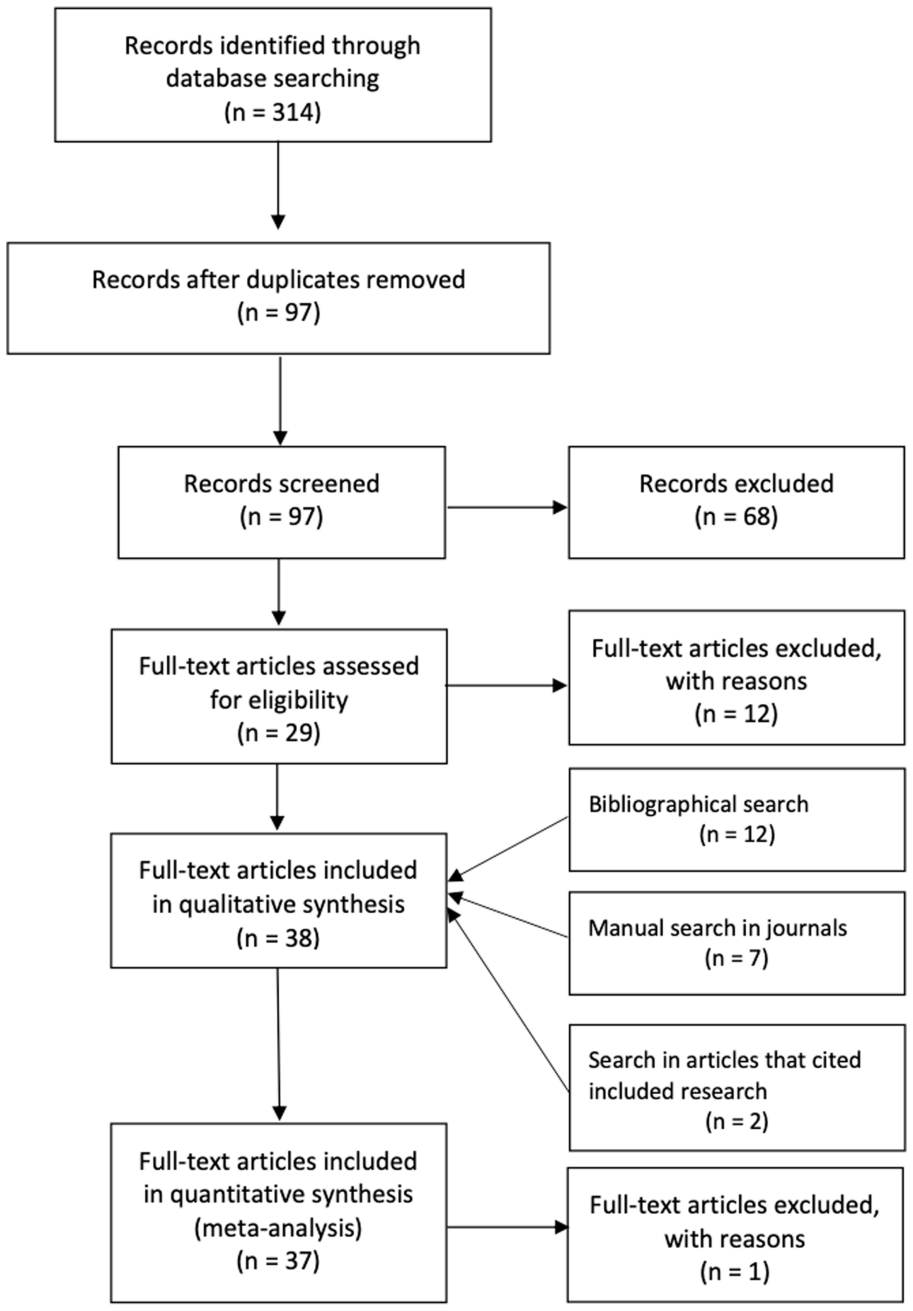
PRISMA flowchart (Moher et al., 2009).

In Figure 2, the PRISMA flowchart is presented (Moher et al., 2009). From the 314 articles returned, 17 were selected, and owing to this small number, to find additional relevant articles, we conducted a search on the reference lists (Horsley et al., 2011) of selected articles, which returned 12 articles that complied with our eligibility criteria (Figure 2). We further performed a manual search in titles and abstracts of 23 journals, when appropriate using the search words “technology” and “work-family,” which returned 7 articles that include variables of after-hours work-related ICT use behaviors. We also extended our search to articles that cited research already included in our search, and this search returned 2 articles. In total, 38 articles are included in the literature review and 37 articles in the meta-analysis.

#### 4.2.1. Meta-analysis coding process

The cumulative inclusion criteria for the meta-analysis, compared to the literature review, consisted in the article presenting the correlations between the study variables of interest to this meta-analysis, namely, work-related after-hours ICT use behaviors and work–family/life conflict, balance, and enrichment. Further, there was a coding of results included in the studies according to the three outcomes variables: work–family/life conflict, balance, and enrichment. In the Supplementary material, all the studies are presented, according to this coding criteria.

## 5. Characterization of studies and qualitative description of results

In this section, the previously mentioned 38 articles resulting from our digital search and screening are reviewed from a qualitative viewpoint. Two of the articles presented two studies (Boswell and Olson-Buchanan, 2007; Ghislieri et al., 2017); thus, this revision included 40 studies in total. All the studies are presented, in a brief summary, in the tables included in the Supplementary material.

As for publication years, the oldest article in our search dates from 1996 (Duxbury et al., 1996) and from 2006 until 2019, 24 more articles were published. However, from 2020 to 2022, our search returned 13 articles. There is an increasing interest in this area, reflected in a rise from an average of 1.8 articles per year, from 2006 to 2019, to an average of 4.3 articles a year from 2020 until 2022 May. This increase underlines the relevance of studying the relationships between work-related after-hours ICT use and work–family/life management variables, which may be in line with recent directives (e.g., Eurofound, 2021) and the development of practical recommendations.

Geographical area representativeness is another concern; our sample reflects some amount of diversity, thereby attending to its small dimension. However, there was an overrepresentation of North American (13) and European (10) articles (Supplementary Table S1). Our search also returned four articles from South Africa, three from China, two from Canada, and one from Taiwan. The remaining five articles did not mention the nationality of participants. Regarding the study design of the 38 articles returned by our search, there was a prevalence of cross-sectional studies (92.11%), and only three articles presented diary studies (Butts et al., 2015; Derks et al., 2015, 2016).

Regarding work–family/life balance, work-related after-hours ICT use showed a negative mediating role in the relationship between social influence and work–family/life balance of teachers (Bauwens et al., 2020), indicating the importance of schools establishing directives restricting the use of after-hours work-related ICT use and therefore reducing the risks of contributing to their teachers’ work–family/life unbalance. In a study by Belkin et al. (2020), the negative relationships between time on email after-hours (considering organizational expectations regarding this behavior as antecedent) and work–family/life balance were positively mediated by work detachment. Organizations must be aware of the negative consequences that the “always on” culture may have on their workers’ well-being, but also individuals must find strategies (such as work detachment) to strive in these fast-paced times (Belkin et al., 2020). Overall, the relationships between work-related after-hours ICT use and work–family/life balance were mainly negative. However, a curvilinear relationship (an inverted-U shape) was found in one of the studies (Chen and Casterella, 2019), indicating that there may exist a less unfavorable side to the use of work-related technology after-hours and work–family/life balance. This study among knowledge workers showed that the flexibility to address some work demands after-hours, granted by ICT use, was favorable up to some point, after which it became detrimental for work–family/life balance. Nevertheless, this research has shown a less unfavorable side of the relationships between work-related after-hours ICT use and work–family/life balance.

Regarding the relationships between after-hours work-related ICT use and work–family/life enrichment, which were analyzed in five studies, the results were more favorable in terms of outcomes related to the work–family/life interface. In a study among workers from a Portuguese service company work-related after-hours ICT use was positively related to work–family/life enrichment, which in turn was related to work engagement (Carvalho et al., 2021). In another research among Italian workers (diverse occupations), only in the male subsample, there was a positive association between work-related after-hours ICT use and work–family/life enrichment, while in the female group, although positive, the relationship was non-significant (Ghislieri et al., 2017). In a third study, among Chinese participants from diverse occupations, the relationships between after-hours work-related ICT use and work–family/life enrichment were also positive; however, there was what the authors called a “double-edged sword effect,” as work–family/life enrichment on its turn was positively related to thriving at work but also to work–family/life conflict (Yang et al., 2022). As for the fifth study, also developed among Chinese workers, although no significant direct relationship was found between after-hours work-related ICT use and work–family/life enrichment, there was a positive mediating effect of work-based resource gain between those variables (Wan et al., 2019).

Regarding the relationships between work-related after-hours ICT use and work–family/life conflict, out of the 35 articles returned in our search, only two reported non-significant direct relationships (Jostell and Hemlin, 2018; van Zoonen et al., 2020). All other studies reported significant positive relationships between work-related after-hours ICT use and work–family/life conflict. Duxbury et al. (1996), in a pioneer study, compared individuals that used and did not use computers to work supplemental hours at home and found that the adopters reported higher levels of work interference with family, as well as role overload and stress, compared to non-adopters. In studies among North American nonacademic managers, work-related ICT use during non-working hours is positively related to work–family/life conflict for individuals (Boswell and Olson-Buchanan, 2007; Diaz et al., 2012) and for their significant others as well (Boswell and Olson-Buchanan, 2007). Also, among academics in a South African higher education establishment, this positive association was found to be moderated by gender, with women reporting higher levels of work–family/life conflict (Kotecha et al., 2014).

Work-related after-hours cell phone use has also shown positive relationships with work–family/life conflict (Brown and Palvia, 2015; Ragsdale and Hoover, 2016; Carlson et al., 2018), among British senior and junior managers (Ward and Steptoe-Warren, 2013), Canadian accountants (Mansour et al., 2022), and work interfering with leisure, which reflected negatively in life satisfaction among Taiwanese workers (Son and Chen, 2018). However, Gadeyne et al. (2018) found no significant relationship between work-related after-hours smartphone use and work–family/life conflict among Flemish working parents of diverse occupations, although this relationship was found to be significant and positive if the used device was a personal computer or laptop. These last results are an important reminder that different technologies may be associated with different impacts on individuals’ work–family/life management.

The great majority of studies found that work-related after-hours ICT use was positively related to work–family/life conflict, such as among Italian workers (Ghislieri et al., 2017), Portuguese company service employees (Carvalho et al., 2021), North American workers (Fenner and Renn, 2010; Harris et al., 2011, 2022; Richardson and Thompson, 2012; Chen and Karahanna, 2014; Wright et al., 2014; Yue, 2022), Canadian workers (Schieman and Young, 2013), and Chinese workers (Wan et al., 2019; Yang et al., 2022) particularly if the interruption is initiated by others compared to initiated by the worker (Khalid et al., 2022). Tams et al. (2020), in a study among North American knowledge workers, showed that perceived work-related after-hours ICT interruption was positively related to work–family/life conflict; however, this effect may be attenuated by workers’ perceptions of control over work. Also, Kim and Hollensbe (2018) in a study among North American information technology workers found that this association could be attenuated by home support. Similarly, Andrade and Petiz Lousã (2021) found in a study among Portuguese workers from diverse occupations that the perceptions of work–family/life conflict associated with work-related after-hours ICT use were favorably moderated by supervisor support and co-worker support.

Three independent studies developed among construction professionals have also shown positive relationships between work-related after-hours ICT use and work–family/life conflict (Bowen et al., 2018; Toland et al., 2020; Zhang and Bowen, 2021), particularly for less experienced workers and women (Bowen et al., 2018).

As for diary studies, daily smartphone use for work purposes after-hours constituted significant work–home interference (Derks et al., 2015); and this effect was stronger when workers preferred to segment, that is, separate work and family/life domains (Derks et al., 2016). Also, the positive relationship between work-related electronic communication during non-work hours and work–family/life conflict was found to be mediated by anger (Butts et al., 2015), in a third diary study.

Gender roles assume significance for understanding work–family/life management (Wood and Eagly, 2010). Kotecha et al. (2014) highlighted the relevance of gender; they found that the positive relationship between technology-assisted supplemental work (TASW) and work–family/life conflict was moderated by gender, with female academics reporting more work–family/life conflict than their male colleagues. Also, these positive relationships between work-related after-hours ICT use and work–family/life conflict were found to be stronger among female construction workers, compared to their male colleagues (Bowen et al., 2018). However, Carvalho et al. (2021), Fenner and Renn (2010), and Ghislieri et al. (2017) did not find significant differences between female and male participants regarding the positive association between after-hours technology-assisted work and work–family/life conflict. In the following meta-analysis the moderating role of gender in the relationship between work-related after-hours ICT use and work–family/life conflict will be tested.

While not addressed in the articles in our review, the COVID-19 pandemics is an essential event that must be acknowledged in this study. Although only four studies had their data gathered during the pandemics, the following meta-analysis includes the moderation test regarding this variable.

## 6. Meta-analytic data analysis

A multilevel meta-analysis was performed using R software (version 4.2.2., R Core Team, 2022), applying the package metafor (Viechtbauer, 2010). Correlations (*r*) were used as effect measures and extracted from pooled studies to be inserted into a random effects model as standardized effect measures based on the inverse variance method, applying Fisher’s to *z* transformation of correlations. Based on the syntax described by Assink and Wibbelink (2016), three-level meta-analytic models were built for “work-family/life enrichment” and “work-family/life conflict” dimensions to account for the dependency of the data. Specifically, in multilevel models, three different sources of variance are modeled: variance between studies (level 3), variance between effect sizes from the same study (level 2), and sample variance between all the effect sizes (level 1). Model coefficients were tested two-sided with the Knapp-Hartung correction (Knapp and Hartung, 2003), and the sampling variance of the effect sizes (level 1) was estimated with Cheung (2014) formula. Heterogeneity was analyzed based on level 2 and/or level 3 significant variance, and subsequent moderation analyses were conducted. Given the small number of effect sizes of the “work-family/life conflict” dimension (none from the same sample), a simple random-effects model was used to pool effect sizes (e.g., Harrer et al., 2021). Potential publication bias was estimated using Duval and Tweedie (2000) trim-and-fill method, which is non-parametric and funnel-plot-based.

## 7. Results

Meta-analytic results for the three work–family/life dimensions are presented in Table 1. Three combined studies revealed a non-significant association between work-related after-hours ICT use and work–family/life balance (*r* = −0.123, *p* = 0.289; 95% CI [−0.353, 0.105]). The average correlation between work-related after-hours ICT use and work–family/life enrichment was pooled from five effect sizes from four studies. This correlation was positive, small, and significant (*r* = 0.124, *p* = 0.013; 95% CI [0.043, 0.206]). Finally, 42 pooled effects from 34 studies were considered to estimate the association between work-related after-hours ICT use and work–family/life conflict. The average effect was positive, moderate in size, and significant (*r* = 0.335, *p* < 0.001; 95% CI [0.290, 0.406]).

**Table 1:** Results for the overall mean effect sizes.

Work–family/life management variables	# Studies	# ES	Fisher’s *z* (SE)	95% CI	Sig. mean z (*p*)	Mean *r*	% Var. level 1	Level 2 variance	% Var. level 2	Level 3 variance	% Var. level 3
Balance	3	3	−0.124 (0.117)	−0.353, 0.105	0.289	−0.123	–	–	–	–	–
Enrichment	4	5	0.125 (0.029)	0.043, 0.206	0.013*	0.124	90.39	0.000	0.00	0.000	9.61
Conflict	34	42	0.348 (0.029)	0.290, 0.406	<0.001***	0.335	9.14	0.004*	14.27	0.021*	76.58

# Studies = number of studies; # ES = number of effect sizes; SE = standard error; CI = confidence interval for Fisher’s z; Sig. mean z = level of significance of mean effect size; Mean r = mean effect size (Pearson’s correlation); % var. = percentage of variance; Level 2 variance = variance between effect sizes within studies; Level 3 variance = variance between studies. * *p* < 0.05; ** *p* < 0.01; *** *p* < 0.001.

The results of the likelihood-ratio tests revealed significant variance within (level 2) and between studies (level 3) in work–family/life conflict dimension. Subsequent moderation analyses (Table 2) revealed a significant effect of the instruments measuring work–family life conflict, *F*(2, 38) = 3.559, *p* = 0.038. Specifically, studies using the Netemeyer et al. (1996) instrument significantly contributed to a greater positive pooled effect (*r* = 0.478, 95% CI [0.376, 0.666]), compared with studies using Carlson et al. (2006) instrument (*r* = 0.295, 95% CI [0.205, 0.403]) or other measures (*r* = 0.312, 95% CI [0.247, 0.398]). Neither pre−/during-COVID period nor gender yielded a significant moderation effect.

**Table 2:** Results for categorical and continuous moderators (bivariate models)—conflict.

Moderators	# Studies	# ES	Intercept (95% CI) / mean *z* (95% CI)	Mean *r*	*β* (95% CI)	*F* (df1, df2)^a^	*p*^b^	Level 2 variance	Level 3 variance
*Time period*						0.214 (1, 39)	0.646	0.004*	0.022*
Pre-COVID (RC)	31	39	0.342 (0.279, 0.406)	0.329					
During COVID	3	3	0.390 (0.191, 0.589)	0.371	0.048 (−0.161, 0.257)				
*Measure of conflict*						3.559 (2, 38)	0.038*	0.004*	0.017*
Netemeyer et al. (1996) (RC)	5	6	0.521 (0.376, 0.666)	0.478					
Carlson et al. (2006)	10	13	0.304 (0.205, 0.403)	0.295	−0.217 (−0.392, −0.042)*				
Others	18	22	0.323 (0.247, 0.398)	0.312	−0.198 (−0.361, −0.035)*				
*% of female participants*	33	41	0.421 (0.278, 0.564)	–	−0.002 (−0.004, 0.001)	1.420 (1, 38)	0.241	0.004*	0.021

# Studies = number of studies; # ES = number of effect sizes; Mean r = mean effect size (r); CI = confidence interval; β = estimated regression coefficient; RC = reference category; Level 2 variance = variance between effect sizes within studies; Level 3 variance = variance between studies. *+p < 0.10*; * p < 0.05; *** p < 0.001.

a Omnibus test of all regression coefficients in the model.

b Value of *p* of the omnibus test.

Inspection of potential publication bias suggested that the previously pooled effect for work–family/life conflict of *r* = 0.335 is underestimated due to small-study effects (Table 3). In the trim-and-fill procedure, 12 “missing” studies were added and a higher bias-corrected effect size was found for this dimension (*r* = 0.382, *p* < 0.001; 95% CI [0.348, 0.455]).

**Table 3:** Results for the overall mean effect sizes after conducting trim and fill analyses.

Work–family/life management variables	# Studies	# ES	Fisher’s *z* (SE)	95% CI	Sig. mean z (*p*)	Mean *r*
Balance	–	–	–	–	–	–
Enrichment	–	–	–	–	–	–
Conflict	46	54	0.402 (0.027)	0.348, 0.455	<0.001	0.382

# Studies = number of studies; # ES = number of effect sizes; SE = standard error; CI = confidence interval for Fisher’s z; Sig. mean z = level of significance of mean effect size; Mean r = mean effect size (Pearson’s correlation). * *p* < 0.05; ** *p* < 0.01; *** *p* < 0.001.

## 8. Discussion

The pervasive nature of ICT use has made it possible for office matters to follow a worker to their home domain and be present beyond working hours. Concerns about the possible effects of the after-hours work-related ICT use are well expressed in a recent European directive on workers’ rights regarding being disconnected and unresponsive to work issues during their leisure time (Eurofound, 2021). This matter has evoked much curiosity, leading to a growing body of empirical literature. However, there is a need to explore the relationships presented in this empirical literature between after-hour work-related ICT use and work–family/life variables, because, to the best of our knowledge, no previous study has examined the global effects—our research aims to bridge this gap. This present study aims to examine the relationships between work-related after-hours ICT use and work–family/life balance, enrichment, and conflict, summarizing the empirical research conducted until May 2022, and analyze the effect sizes of the relationships between these variables, as well as the potential role of gender and pre/during COVID-19 data gathering in these relationships.

The association between work-related after-hours ICT use and work–family/life balance was not significant. The balance between work and family/life domains is conceptualized as an equilibrium between expectations, negotiated and shared between the persons involved in those domains (Grzywacz and Carlson, 2007); therefore, work-related after-hours ICT use may present challenges to this equilibrium, tipping the scales toward the work domain. However, the results of our study are not in line with research that indicates that work-related after-hours ICT use interferes negatively with work–family/life balance, for example, leading workers to perceive less autonomy in managing the interface between these two domains (Wang et al., 2020). This non-significant result may reflect the scarce number of studies and the fact that one showed an inverted-U shape in the relationships between work-related after-hours ICT use and work–family/life balance, reflecting also the flexibility advantages given by the use of technology. That is, in the right measure work-related after-hours technology use may contribute to work–family/life balance (e.g., allowing to reply to that urgent email from the boss), but when it becomes too invasive its negative impact prevails (e.g., when one email turns into a time-consuming digital conversation with the supervisor).

A positive and significant association was found between work-related after-hour ICT use and work–family enrichment. However, owing to the range of heterogeneity, this positive relationship requires a cautious analysis and calls for further research to establish a more reliable result that reflects the association between these variables. Therefore, our results must be interpreted with caution owing to the small number of studies and heterogeneity of measures. In fact, only three studies have researched the associations between work-related after-hours ICT use and work–family balance, and five have researched the associations between the former and work–family/life enrichment. This lack of research on the relationships between work-related after-hours ICT use and positive aspects of the work–family/life interface seems to reflect a negative bias; however, it is significant to study the potential contributions from a positive perspective, particularly regarding work–family/life enrichment, and the double-edged sword character referred to by certain authors (e.g., Yang et al., 2022). Moreover, ICT use contributes to more flexibility and skill acquisition that can translate into resources, which may be transferred to the family/life domain, enhancing the positive effects of these tools.

According to Greenhaus and Beutell (1985), three major sources contribute to work–family/life conflict: time allocated to the job, strain associated with the work role, and specific behaviors required by the job role, which interfere negatively in fulfilling the family/life role. Work-related after-hours ICT use has the potential to present, if not all, at least one of these negative characteristics. Therefore, as expected, this study shows that the relationship between work-related after-hours ICT use and work–family/life conflict is significant and positive—although moderate—indicating that it is a source of conflict between these two domains. Owing to high heterogeneity, moderation effects of work–family/life conflict measures used in the studies showed that the scale developed by Netemeyer et al. (1996) revealed a tendency for the pooled effect to be greater than the studies using other measures. In the 34 studies that investigated work–family/life conflict and were included in the meta-analysis, 12 different scales were reported. However, all scales reflected the same negative trend in the relationship between work-related after-hours ICT use and work–family/life conflicts.

There were no differences found between the studies that collected data before and during the COVID-19 pandemics, which seems to indicate that the alterations brought on by this event do not reflect significantly in the relationships between work-related after-hours ICT use and the work–family/life interface.

Regarding the possible effects of different roles assumed by men and women in our society in the work and home domains (Wood and Eagly, 2010), the effect of gender on work–family/life was tested. However no moderating effect was found. In a previous meta-analysis, little significant differences were found between male and female perceptions of work–family/life conflict (Shockley et al., 2017). This absence of significant differences between women and men regarding work–family/life conflict may reflect a rising attenuation of the differentiation of gender roles. Albeit recent surveys show a certain regression in attitudes toward gender equality (UN Women, 2022), these may not be reflected in the studies included in our research.

## 9. Limitations and future research

Our study had a few limitations. First, it examined a small number of studies that restrained the analyses and affected their degree of precision. Additionally, the high heterogeneity of the measures used in the studies did not contribute to the solidity and precision of our results.

Regarding eligibility criteria, only peer-reviewed articles were included (excluding all non-peer-reviewed literature and gray literature). This decision may have introduced bias in the findings; however, our publication bias analysis showed that this bias may not be present in our data (Duval and Tweedie, 2000), and our criteria reflected the need to ensure quality of the research.

While we aimed to study the relationships between work-related after-hours ICT use and positive effects, such as work–family/life balance and enrichment, our search returned only a few studies. Future studies should explore these positive aspects, for work-related ICT use may be associated with more flexibility and skill transfer between domains. The inclusion of boundary theory variables, such as perceived control and segmentation preferences/reported behavior, may contribute to research on moderators and mediators in these relationships.

## 10. Theoretical, methodological, and practical implications

Our research shows positive relationships between after-hours work-related technology use and work–family/life conflict, however also with work–family/life enrichment. Furthermore, there was no significant relationship between after-hours work-related technology use and work–family/life balance. This study contributes to work–family/life conflict literature, underlining the fact that after-hours work-related technology use has a negative effect, contributing to workers’ perceptions of conflict between work and family/life, which previous literature has shown to negatively relate to individuals’ well-being (Baumeister et al., 2021). Previous meta-analysis have shown that resource depletion due to long working hours (Allen et al., 2020) and work role overload and time demands (Michel et al., 2011) are antecedents of work–family/life conflict. Our study further indicates that after-hours work-related technology also contributes to work–family/life conflict. This result is particularly relevant in these times when telecommuting seems to be a post-COVID-19 heritage, which bring benefits (such as flexibility) but also due to the pervasive ICT use may lead “to longer working hours and the blurring of boundaries between work and private life” (Eurofound, 2021, p: 1). Therefore, to prevent the negative effects of a culture of “always on” organizations are advised to contribute to their workers’ beliefs in the right to disconnect, following the empirical evidence that also translated into official directives (Eurofound, 2021).

Work–family/life interface shows once more its complexity, which translates into the same variable showing seemingly contradictory effects, such as after-hours work-related technology use and positive associations with work–family/life enrichment. Also, its association with work–family/life balance, according to our findings, is not always significantly negative. In order to further explore this complexity, future research should include diverse moderator variables. For example, the workers’ volition regarding the after-hours work versus the organizational imposition to work after-hours, may better explain the positive work–family/life enrichment association, and the negative work–family/life conflict relationship. Also, the degree to which the worker perceives after-hours work-related technology use as flexibility to manage their working schedule, and not an imposed burden that uses up family/personal-life time, may contribute to a less negative association between this variable and work–family/life balance.

As for our methodological contributions, although there is a high heterogeneity of measures of work–family/life conflict, our results indicate that while all instruments adequately measure this construct, the scale developed by Netemeyer et al. (1996) shows stronger positive relationships with work-related after-hours ICT use, compared to other measures, and may be considered better adjusted to assess this variable.

Our study also showed no significant differences in the relationships between work-related after-hours ICT use and work–family/life conflict between women and men, as in a previous meta-analytical study (Shockley et al., 2017), underlining that work–family/life interface is important irrespective of gender. Organizations, therefore, should continue to develop gender-equality policies and practices. Also, as noted previously, our study may not reflect a certain reversion in attitudes toward gender equality captured in a recent survey (UN Women, 2022), so future research should not neglect possible gender differences in the work–family/life interface.

Work-related after-hours ICT use seems to be a practice that has become increasingly relevant in our times, due to its growing presence and potential negative effects. Consequently, organizations should consider refraining from imposing this practice to its working force, as it can lead to negative outcomes, such as higher levels of work–family/life conflict and ill-being. However further research is needed to explore the situations where this practice may be useful and bring positive outcomes to the worker as well as to the organization.

## Author contributions

AS: study design and execution, data analysis, and writing, editing, and revising of the manuscript. CC: data analysis, and writing, editing, and revising of manuscript. MR: data analysis, and writing and editing of manuscript. MC: study design, data analysis, and writing, editing, and revising of the manuscript. All authors contributed to the article and approved the submitted version.

## Funding

The authors acknowledge financial support from FCT - Fundação para a Ciência e a Tecnologia, I.P., granted to the project “Work -Family boundary dynamics in nontraditional jobs” (PTDC/PSI-GER/32367/2017). This work also received national funding for publication from FCT - Fundação para a Ciência e a Tecnologia, I.P., through the Research Center for Psychological Science of the Faculty of Psychology, University of Lisbon (UIDB/04527/2020;UIDP/04527/2020).

## Conflict of interest

The authors declare that the research was conducted in the absence of any commercial or financial relationships that could be construed as a potential conflict of interest.

## Publisher’s note

All claims expressed in this article are solely those of the authors and do not necessarily represent those of their affiliated organizations, or those of the publisher, the editors and the reviewers. Any product that may be evaluated in this article, or claim that may be made by its manufacturer, is not guaranteed or endorsed by the publisher.

## Footnotes

^1^We opted for the designation used by Kossek et al. (2021) of work–family/life, in need of a broader concept compared to work–family and also to avoid the current proliferation of designations.
