# Supplementary material for: Information and communication technologies-assisted after-hours work: A systematic literature review and meta-analysis of the relationships with work–family/life management variables
Source: Front Psychol. 2023 Feb 1;14:1101191. doi: 10.3389/fpsyg.2023.1101191 (PMC9928856; doi:10.3389/fpsyg.2023.1101191)
Supplement: Supplementary file 1 [file Table_1.pdf]

Supplementary material

Table 1. Studies according to geographical area

Africa	Asia	Canada	Europe	USA	No mention
Bowen et al. (2018) South Africa	Khalid et al. (2021) China	Mansour et al. (2021)	Andrade & Lousã (2020) Portugal	Belkin et al. (2020)	Boswell & Olson-Buchanan (2006)
Kotecha et al. (2014) South Africa	Son & Chen (2018) Taiwan	Schieman & Young (2013)	Bawens et al. (2020) Belgium	Butts et al. (2015)	Brown & Palvia (2015)
Toland et al. (2020) South Africa	Wan et al. (2019) China		Carvalho et al. (2021) Portugal	Chen & Casterella (2019)	Carlson et al. (2018)
Zhang & Bowen (2021) South Africa	Yang et al. (2022) China		Derks et al. (2015) Netherlands	Chen & Karahana (2014)	Duxbury et al. (1996)
			Derks et al. (2016) Netherlands	Diaz et al. (2012)	Ragsdale & Hoover (2016)
			Gadeyne et al. (2018) Flanders/ Belgium	Fenner & Renn (2010)	
			Ghislieri et al. (2017) Italy	Harris et al. (2011)	
			Jostell & Hemlin (2018) Sweeden	Harris et al. (2021)	
			van Zoonen et al. (2020) Scandinavia	Kim & Hollensbe (2018)	
			Ward & Steptoe-Warren (2013) Great Britain	Richardson &Thompson (2012)	
				Tams et al. (2020)	
				Wright et al. (2014)	
				Yue (2022)	

Table 2. Studies including work-family/life balance

		Sample						Variables and measures			
**Author(s) Country**	**Year**	**Dimension**	**Pre-COVID-19**	**During COVID-19**	**Gender (female)**	**Occupation**	**Design**	**After-hours ICT use**	**Measure**	**Outcome**	**Measure**
Bawens et al. Belgium	2020	N=288	x		64,3%	Secundary school teachers	Cross-sectional	Work-related ICT use after hours	Based on Boswell and Olson-Buchanan (2007) and Richardson and Benbunan-Fich (2011)	Work-life balance	Valcour (2007)
Belkin et al. USA	2020	N=563	x		36%	Diverse	Cross-sectional (two surveys, one week)	Time spent on work email on non-work time	Estimated time, in hours, during a typical week	Work-life balance	Five-item scale from Allen and colleagues (2010)
Chen & Casterella	2019	N=312	x		53%	Knowledge workers	Cross-sectional	After-Hours Work-Related Technology Use	Authors "asked respondents to identify the devices and applications that they typically used for after-hours work engagement." p. 83	Work-life balance	Carlson et al., 2009

Table 3. Studies including work-family/life enrichment

		Sample						Variables and measures			
**Author(s) Country**	**Year**	**Dimension**	**Pre-COVID-19**	**During COVID-19**	**Gender (female)**	**Occupation**	**Design**	**After-hours ICT use**	**Measure**	**Outcome**	**Measure**
Carvalho et al. Portugal	2021	N=533	x		47,3%	Service company	Cross-sectional	Technology-assisted supplemental work (TASW)	Based on Ghislieri et al. (2017)	Work-family enrichment	Nine-item scale (Carlson et al., 2006)
Ghislieri et al. Italy	2017	N=352	x		52,5%	Diverse	Cross-sectional	Off-work hours technology assisted job demand	Three ad hoc items	Work-family enrichment	Three items (Ghislieri et al., 2011)
Ghislieri et al. Italy	2017	N=319	x		male sample	Diverse	Cross-sectional	Off-work hours technology assisted job demand	Three ad hoc items	Work-life enrichment	Three items (Ghislieri et al., 2011)
Wan et al. China	2019	N=111	x		51%	Diverse	Cross-sectional (two wave-study)	Cross-domain work communication	Items developed by authors	Work-to-family enrichment	Three-item scale (Kacmar et al., 2014)
Yang et al. China	2022	N=257		x	53%	Diverse	Cross-sectional. (Two data collecting points, two-week lag)	Work connectivity behavior after-hours	Six-item scale (Fenner & Renn, 2010)	Work-family enrichment	Nine-item scale (Carlson et al.,2006)

Table 4. Studies including work-family/life conflict

		Sample						Variables and measures			
Author(s)<br><br>Country	Year	Dimension	pre-COVID-19	During COVID-19	Gender (female)	Occupation	Design	After-hours ICT use	Measure	Outcome	Measure
Andrade & Lousã<br><br>Portugal	2020	N=212		x	71,7%	Diverse	Cross-sectional	After hours work-related technology use	Adapted version of the scale develeoped by Piszczek, 2017	Work-family conflict	Three-item scale adapted from Mathews et al., 2011
Boswell & Olson-Buchanan	2006	N=360,	x		67%	Nonacademic staff positions at university;	Cross-sectional	Communication technologies use after hours	Batt and Valcour's (2003) adapted measure of flexible technology use	Work-life conflict	Gutek et al.'s (1991) four-item Work Interference With Family Scale
Boswell & Olson-Buchanan	2006	N=35 significant others	x		Non available	Significant others of managers and administrators	Cross-sectional	Communication technologies use after hours	Batt and Valcour's (2003) adapted measure of flexible technology use	Work-life conflict	Gutek et al.'s (1991) four-item Work Interference With Family Scale
Bowen et al.<br><br>South Africa	2018	N=630	x		18%	Construction professionals	Cross-sectional	Work contact	Three items from Schieman and Young (2013)	Work-life conflict	Four items from Voydanoff (2007) and Schieman and Young (2013)

Table 4. Studies including work-family/life conflict (cont.)

		Sample						Variables and measures			
Author(s)<br><br>Country	Year	Dimension	pre-COVID-19	During COVID-19	Gender (female)	Occupation	Design	After-hours ICT use	Measure	Outcome	Measure
Brown & Palvia	2015	N=165	x		45%	Diverse	Cross-sectional	Mobile device usage (work-related) at home	Non available	Work-life conflict	Non available
Butts et al.	2015	N=341 (N=1572 daily surveys)	x		36%	Diverse	Daily surveys (seven)	Work-related electronic communication during non-work times	Responses coded in hours and fractions of hours	Work-to-nonwork conflict	5-item scale (Netemeyer et al., 1996 - adaptated to a nonwork domain and not just family domain)
Carlson et al.	2018	N= 344 pairs of workers and spouses	x		39%	Diverse	Cross-sectional	Mobile device use for work during family time	Three-item scale (Ferguson et al., 2016)	Work-to-family conflict	Nine-item scale (Carlson et al.,2000)
Carvalho et al.<br><br>Portugal	2021	N=338	x		47,3%	Service company employees	Cross-sectional	Technology-assisted supplemental work (TASW)	Three items developed by Ghislieri et al. (2017)	Work-family conflict	10 items (Carlson, Kacmar, & Williams, 2000)

Table 4. Studies including work-family/life conflict (cont.)

		Sample						Variables and measures			
**Author(s)**<br><br>**Country**	**Year**	**Dimension**	**pre-COVID-19**	**During COVID-19**	**Gender (female)**	**Occupation**	**Design**	**After-hours ICT use**	**Measure**	**Outcome**	**Measure**
Chen & Karahana<br><br>USA	2014	N=137	x		59,4%	Technology firm workers	Cross-sectional	Frequency of work to nonwork (WTN) interruptions	Items created by authors	Work to nonwork conflict	Three-item scale (Netemeyer et al., 1996)
Derks et al.<br><br>Netherlands	2016	N=71	x		44%	Diverse	Diary study, 4 days, 265-280 data points	Work-related smartphone use during off-work time	Four- item smartphone use scale developed by Derks and Bakker (2014a)	Work-family conflict	Five-item subscale (Netemeyer et al., 1996)
Derks et al.<br><br>Netherlands	2015	100 (367-400 data points)	x		25%	Diverse	Four-day diary study	Daily smartphone use after working hours	Four-item smartphone use scale (Derks & Bakker, 2014)	Daily work-home interference	Eight-item subscale of the Survey Work-home Interaction NijmeGen (Geurts et al., 2005)
Diaz et al.<br><br>USA	2012	N=193	x		45%	Non-academic university managers	Cross-sectional	Communication technology use	Two items developed by authors	Work-to-life conflict	Four-item scale (Gutek et al., 1991)
Duxbury et al. (*)	1996	N=454	x		32,38%	Diverse	Cross-sectional	Computer-supported supplemental work-at-home	Average time using computer for after-hours work-related tasks	Work-family conflict (interference)	Bohen and Viveros-Long (1981)

Table 4. Studies including work-family/life conflict (cont.)

		Sample						Variables and measures			
**Author(s)**<br><br>**Country**	**Year**	**Dimension**	**pre-COVID-19**	**During COVID-19**	**Gender (female)**	**Occupation**	**Design**	**After-hours ICT use**	**Measure**	**Outcome**	**Measure**
Fenner & Renn<br><br>USA	2010	N=227	x		28%	Diverse	Cross-sectional	Technology-assisted supplemental work (TASW)	six-item scale developed by authors.	Work-family conflict	three items from Kopelman et al.'s (1983); two items from the Frone et al. (1992)
Gadeyne et al. Belgium	2018	N=467	x		85%	Diverse (working parents)	Cross-sectional	Smartphone use	Two indicators: how often and average minutes	Time-based work-to-home conflict	Three-item scale (Carlson et al., 2000)
Gadeyne et al. Belgium	2018	N=467	x		85%	Diverse (working parents)	Cross-sectional	Smartphone use	Two indicators: how often and average minutes	Strain-based work-to-home conflict	Three-item scale (Carlson et al., 2000)
Gadeyne et al. Belgium	2018	N=467	x		85%	Diverse (working parents)	Cross-sectional	Pc/laptop use	Two indicators: how often and average minutes	Time-based work-to-home conflict	Three-item scale (Carlson et al., 2000)
Gadeyne et al. Belgium	2018	N=467	x		85%	Diverse (working parents)	Cross-sectional	Pc/laptop use	Two indicators: how often and average minutes	Strain-based work-to-home conflict	Three-item scale (Carlson et al., 2000)

Table 4. Studies including work-family/life conflict (cont.)

		Sample						Variables and measures			
**Author(s)**<br><br>**Country**	**Year**	**Dimension**	**pre-COVID-19**	**During COVID-19**	**Gender (female)**	**Occupation**	**Design**	**After-hours ICT use**	**Measure**	**Outcome**	**Measure**
Ghislieri et al.<br><br>Italy	2017	N=352	x		52,5%	Diverse	Cross-sectional	Off-work hours technology assisted job demand	Three ad hoc items	Work-family conflict	Five items of the Netemeyer et al. (1996) scale
Ghislieri et al.<br><br>Italy	2017	N=319	x		male sample	Diverse	Cross-sectional	Off-work hours technology assisted job demand	Three ad hoc items	Work-life conflict	Five items of the Netemeyer et al. (1996) scale
Harris et al.	2021	N=253			62%	Diverse	Cross-sectional (Two data collecting points, six weeks time lag)	Techno-invasion	Three-item scale (Ragu-nathan et al., 2008) (Sample item: "I feel my personal life is being invaded by ICTs", p. 1080)	Work-family conflict	Three-item short form scale (Carlson et al., 2000)
Harris et al.<br><br>USA	2011	N=283	x		59%	Diverse	Cross-sectional	Technology related pressure	Five items developed by authors	Work-family conflict	18-item scale (Carlson et al., 2000)
Jostell & Hemlin<br><br>Sweeden	2018	N=71	x		35%	Sales department employees	Cross-sectional	After hours teleworking	Item developed by authors	Work-life conflict (work interference with family)	Eight-item measure by Gutek et al. (1991)

Table 4. Studies including work-family/life conflict (cont.)

		Sample						Variables and measures			
**Author(s)**<br><br>**Country**	**Year**	**Dimension**	**pre-COVID-19**	**During COVID-19**	**Gender (female)**	**Occupation**	**Design**	**After-hours ICT use**	**Measure**	**Outcome**	**Measure**
Khalid et al.<br><br>China	2021	N=318	Non available		47,8%	Diverse	Cross-sectional. (Three data collecting moments, six week-lag each)	After hours work-related technology use	Five-item scale adapted from Boswell and Olson-Buchanan (2007) and Batt and Valcour (2003)	Work-family conflict	Five-item scale (Stephens & Sommer, 1996)
Kim & Hollensbe<br><br>USA	2018	N=267	x		27,7%	Information technology workers	Cross-sectional (Two data collecting points, two weeks time lag)	Technology-related pressure	Five-item scale (Harris et al., 2011)	Work-family conflict	Six-item scale (Carlson et al., 2000)
Kotecha et al.<br><br>South Africa	2014	N=216	x		60,2%	Academics in higher education	Cross-sectional	Technology- assisted supplemental work (TASW)	Six items (Fenner & Renn, 2010)	Work-life conflict	Nine items (Carlson, Kacmar, & Williams, 2000)
Mansour et al.<br><br>Canada	2021	N=388	x		67%	Accountanting occupations	Cross-sectional	Smartphone and/or tablet use	Five-item scale (Derks et al., 2014)	Work-family conflict	Five items adapted by authors
Ragsdale & Hoover	2016	N=313	x		52%	Diverse	Cross-sectional (Two data collecting points, one week time lag)	Work-related cell phone use	Developed by authors (Sample item: "I find myself using my cell phone for work at home", p. 57)	Work-family conflict	Six-item scale (Carlson et al., 2000)

Table 4. Studies including work-family/life conflict (cont.)

		Sample						Variables and measures			
**Author(s)**<br><br>**Country**	**Year**	**Dimension**	**pre-COVID-19**	**During COVID-19**	**Gender (female)**	**Occupation**	**Design**	**After-hours ICT use**	**Measure**	**Outcome**	**Measure**
Richardson &Thompson	2012	N=139	x		69%	Diverse	Cross-sectional	Work connectivity behaviour after-hours - duration	Items developed by authors measuring frequency and duration	Work-family conflict	Eight-item scale (Kopelman et al., 1983)
Richardson &Thompson<br><br>USA	2012	N=139	x		69%	Diverse	Cross-sectional	Work connectivity behaviour after-hours - frequency	Items developed by authors to measure duration and frequency	Work-family conflict	Eight-item scale (Kopelman et al., 1983)
Schieman & Young<br><br>Canada	2013	N=5729	x		48%	Diverse	Cross-sectional	Work contact	Three-item scale	Work-family conflict	Four-item scale (e.g., Schieman & Glavin, 2011)
Son & Chen<br><br>Taiwan	2018	N=462	x		51,7%	Diverse	Cross-sectional	Daily smartphone use	(Derks & Bakker, 2014)	Work-leisure conflict: time-based<br><br>strain-based	(Tsaur et al., 2012)
Tams et al.<br><br>USA	2020	N=601	x		45%	Knowledge workers	Cross-sectional	Perceived interruption overload	Four items adapted from Chen and Karahanna, 2018; Yin et al., 2018	Work-life conflict	Five items adapted from Adams et al., 1996; Ahuja et al., 2007; Netemeyer et al., 1996, 2004

Table 4. Studies including work-family/life conflict (cont.)

		Sample						Variables and measures			
**Author(s)**<br><br>**Country**	**Year**	**Dimension**	**pre-COVID-19**	**During COVID-19**	**Gender (female)**	**Occupation**	**Design**	**After-hours ICT use**	**Measure**	**Outcome**	**Measure**
Toland et al.<br><br>South Africa	2020	N=864	x		Not reported	Construction professionals	Cross-sectional	Work contact after hours	Three items adapted from Schieman and Young (2013)	Work-life conflict	Four items drawn from Schieman and Young (2013)
van Zoonen et al.<br><br>Scandinavia	2020	N=367	x		45,1%	Knowledge workers	Cross-sectional (two surveys one year apart)	Smartphone use outside formal work hours	Three items developd by authors	Work-life conflict	Four items adopted from Hayman (2005)
Wan et al.	2019	N=111	x		51%	Diverse	Cross-sectional (two wave-study)	cross-domain work communication	items developed by authors	Work-family conflict	Four-item scale (Grzywacks & Marks, 2000)
Ward & Steptoe-Warren<br><br>Great Britain	2013	N=86	x		24,4%	Junior and senior managers	Cross-sectional	Blackberry use for work porpuses during non-working hours	Work Connectivity Behaviour After-Hours (WCBA) duration measure (Richardson & Thompson, 2012).	Work-family conflict	Eight item scale (Kopelman et al., 1983)

Table 4. Studies including work-family/life conflict (cont.)

		Sample						Variables and measures			
**Author(s)**<br><br>**Country**	**Year**	**Dimension**	**pre-COVID-19**	**During COVID-19**	**Gender (female)**	**Occupation**	**Design**	**After-hours ICT use**	**Measure**	**Outcome**	**Measure**
Wright et al.<br><br>USA	2014	N=168	x		66%	Diverse	Cross-sectional	Work-related communication technology use outside of regular work hours	Form of communication technology used, and estimation of weekly average time spent using it for after-hours work-related purposes	Work-life conflict	Seven-item scale (modified version of Hayman's 2005 work-life balance scale
Yang et al.<br><br>China	2022	N=257		x	53%	Diverse	Cross-sectional. Two data collecting points, two-week lag	Work connectivity behavior after-hours	Six-item scale (Fenner & Renn, 2010)	Work-family conflict	Nine-item scale (Carlson et al., 2000).
Yue<br><br>USA	2022	N=815		x	54%	Diverse	Cross-sectional	Work-related social media use	Participants were asked to reference their behaviors over the last 30 days, on the four scales.	Work-family conflict	Six-item scale (Carlson et al., 2000)
Zhang & Bowen<br><br>South Africa	2021	N=851	x		18%	Construction industry workers	Cross-sectional	Work contact	Schieman and Young (2013); Bowen et al. (2018)	Work-family conflict	Schieman and Young (2013); Bowen et al. (2018)

Note – all studies, except for Duxbury et al. (1996) (*) were included in the meta-analysis.
